# Morphological consistency of bilateral hip joints in adults based on the X-ray and CT data

**DOI:** 10.1007/s00276-020-02676-4

**Published:** 2021-01-23

**Authors:** Ran Zhao, Hong Cai, Hua Tian, Ke Zhang

**Affiliations:** Department of Orthopaedic Surgery, Peking University Third Hospital, NO. 49 North Gardon Road, Haidian District, Beijing, China

**Keywords:** Anatomy, Hip anatomy, Coxometry, Symmetry, Total hip arthroplasty

## Abstract

**Purpose:**

The application of the anatomical parameters of the contralateral hip joint to guide the preoperative template of the affected side relies on the bilateral hip symmetry. We investigated the bilateral hip symmetry and range of anatomical variations by measurement and comparison of bilateral hip anatomical parameters.

**Methods:**

This study included 224 patients (448 hips) who were diagnosed with osteoarthritis (OA) and avascular necrosis (AVN) of the femur head, and underwent bilateral primary total hip arthroplasty (THA) in our hospital from January 2012 to August 2020. Imaging data included 224 patients X-ray and 30 CT data at the end of the cohort. Anatomical parameters, including the acetabular abduction angle and trochanteric height, were measured using the Noble method. Postoperative measurements included stem size, difference of leg length and offset.

**Results:**

Except for the isthmus width, there were no significant differences in the anatomical morphology of the hip joint. Among the demographic factors, there was a correlation between body weight and NSA. Among various anatomical parameters, a correlation was present between medullary cavity widths of T + 20, T, and T − 20. The difference in the use of stem size is not due to the morphological difference of bilateral medullary cavity, but due to the different of 1- or 2-stage surgery.

**Conclusion:**

Bilateral symmetry was present among the patients with normal morphology of the hip medullary cavity, theoretically confirming the feasibility of structural reconstruction of the hip joint using the hip joint on the uninjured side. Additionally, the difference in the morphology of the hip medullary cavity is not present in a single plane but is synergistically affected by multiple adjacent planes.

## Introduction

Total hip arthroplasty (THA) effectively relieves pain in the hip joints and restores hip joint function. The success of hip arthroplasty depends on accurate matching of the prosthesis with the morphology of femur and the acetabulum, which also promotes the development of morphological studies of the femoral medullary cavity. When the proximal femur is deformed by degeneration, such as femoral neck fracture, hip dysplasia, coxa plana and similar conditions, measurements of the normal anatomy often become difficult. At present, the commonly used method is to measure the template of THA through the contralateral hip joint, to restore the leg length and offset [18]. However, this method is based on the bilateral symmetry of the lower limbs. Previous studies have focused on the bilateral femoral asymmetry, but studies on asymmetry have mainly concentrated on bone density changes, mechanical strength, the rotation angle, the lower limb length, and the distal femur [2, 8]; very few reports have focused on the morphological symmetry of the proximal femur. The purposes of this study were: first, to compare the bilateral symmetry of the bilateral acetabular and proximal femoral medullary morphology by preoperative template measurement in patients undergoing bilateral hip arthroplasty; second, to measure the range of normal values of bilateral hip joint differences and their relationship with body weight, age, and sex; third, to study the range of the normal variations of bilateral anatomical differences; and fourth, to explore the correlation of variations between anatomical parameters.

## Materials and methods

### Study cohort

This study involved a cohort of patients who were diagnosed with primary osteoarthritis and avascular necrosis of the femoral head from January 2012 to August 2020 and underwent 1-stage or 2-stage bilateral THA. All patients had complete preoperative medical records as well as positive and lateral hip joint radiographs and pelvic anteroposterior radiographs of the injured limbs. The patients were selected for the study by six senior physicians, and each patient underwent the operation by the same physician either 1-stage or 2-stage. We excluded patients with diseases affecting the morphology or bone mass of the proximal femoral medulla, such as developmental dysplasia of the hip (DDH), a history of long-term steroid use, a history of proximal femoral osteotomy or internal fixation, or a history of bone tumors in the proximal femur; we also excluded patients with incomplete imaging data and non-standardized films.

In total, 224 patients (135 males and 89 females) were included. At the time of the operations, the average age of the patients was 53.8 ± 11.1 years (range, 21–88 years), and the cohort comprised 135 men and 89 women. The mean body weight was 70.3 ± 11.8 kg (range, 44–114 kg), and the mean body mass index was 25.3 ± 3.5 kg/m^2^ (range, 17.3–37.2 kg/m^2^).

In the last 30 patients in this cohort (9 males and 31 females), we also took computed tomography (CT) before surgery. The average age of these 30 patients was 52.7 ± 10.9 years.

### X-ray parameters

Radiographic films were taken in a standardized way as follows. The patients lay on the camera, the distance from the device was 100 cm, and the X-ray of the hip joint in the positive and lateral position was centered on the level of the lesser trochanter. The femur was rotated to place the femoral neck in the coronal plane. For the pelvic anteroposterior radiographs, the pelvis was positioned in the horizontal line along the pubic symphysis (the same line as the midline of the sacrum), and the big toes of each foot were touching each other to maintain consistent femoral rotation.

### CT parameters

All participants underwent 64-slice CT scanning (Discovery 750 HD; GE Healthcare, Madison, WI, USA) using the same projection protocol. The parameters were: 120 kV; 80 mAs; reconstruction thickness, 1.0 mm; interlayer spacing, 1.0 mm. Each participant was placed in supine position with the lower limbs fixed in a neutral position, fully extended and slightly rotated, with the toes pointing upward. The lower limbs were fixed, and seat belts were fastened to prevent movement during the CT process. Participants were scanned from the iliac crest to the knee joint. The images were saved as DICOM images.

### Measurements

**Fig. 1:**
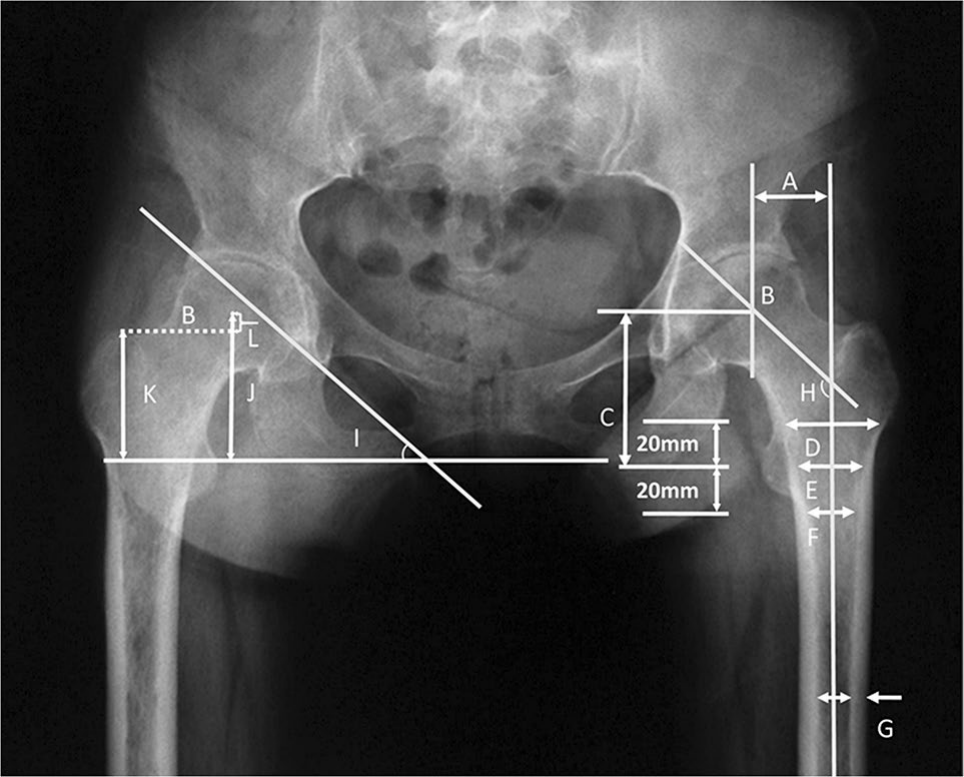
Radiologic measurements of the proximal femur according to Noble et al. [12]. **a** Femoral head offset. **b** Center of the femoral head. **c** Femoral head position. **d** Cavity width 20 mm above the mid-lesser trochanter line (T + 20). (E) Cavity width at the mid-lesser trochanter line (T + 0). **f** Cavity width 20 mm below the mid-lesser trochanter line (T − 20). **g** Isthmus diameter. **h** Neck-shaft angle. The canal flare index defined by Noble et al. [4] was D/G. (I) Acetabular abduction angle. **k** Vertical distance between the tip of the greater trochanter and the ischial tuberosity line. **j** Vertical distance between the center of the femoral head and the ischial tuberosity line; the difference in the vertical distance L of the greater trochanter was **j** − **k**

A picture archiving and communication system was used in every case, and the Noble classification was used to evaluate the anatomy and morphologic features of the proximal femur [12] (Fig. 1). Noble et al. [12] used the canal flare index (CFI) to describe the shape of the proximal femur, which was defined as the ratio of 20 mm (T + 20) above the mid-lesser trochanter line and medullary isthmus (isthmus). We also measured the acetabular abduction angle and the trochanteric height, which was defined as the height of the greater trochanter relative to the center of the femoral head (Fig. 1).

**Fig. 2:**
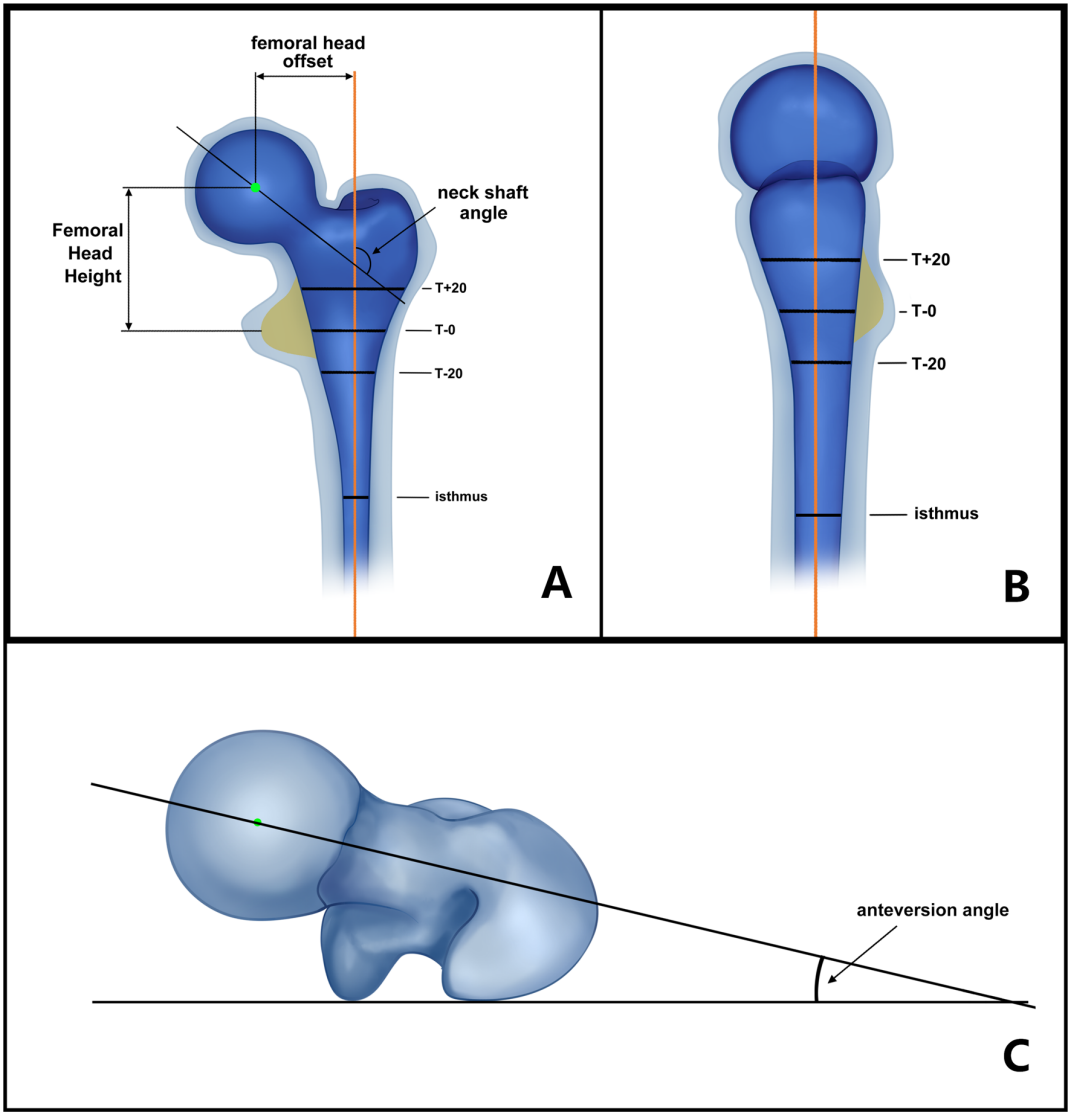
Femoral medullary cavity measurement parameters of 3D data measurement. **a** Coronal plane parameters. **b** Sagittal plane parameters. **c** Anteversion angle

In terms of CT data, in addition to the femoral medial–lateral (ML) data similar to X-ray measurements, we also measured the sagittal anterior–posterior parameter (AP) and anteversion of the femur (Fig. 2).

According to the postoperative medical records, the date of bilateral operation (1-stage group or 2-stage group) was recorded. The consistency of bilateral prosthesis was compared between the two groups. We also measured the bilateral femoral offset and the difference of leg length after finishing bilateral surgery.

The radiologic measurements were reanalyzed by the same observer (R.Z.) to determine the intraobserver agreement and by another observer (H.C.) to determine the interobserver agreement. All anatomical parameters were measured again 1 month later.

### Statistical analysis

The Kolmogorov–Smirnov test was used to test the normality of the data and showed that the data exhibited a normal distribution. The paired-sample *t* test was used to calculate the symmetry of the left- and right-limb anatomical parameters, the symmetry of the left- and right-limb anatomical parameters of the stem-side inconsistency group. The independent *t* test was used to calculate the statistical difference in the absolute differences (AB) between the stem-side inconsistency group and the stem-side consistency group, and between the gender groups. A *P* value of < 0.05 was considered statistically significant. Correlation analysis was used to study the correlations between AB in anatomical parameters and sex, age, height, and weight as well as the correlations between anatomical parameters. The intragroup correlation coefficient was used to evaluate the intraobserver and interobserver agreements.

All statistical analyses were performed with SPSS version 20.0 (IBM Corp., Armonk, NY, USA).

## Results

The intraobserver and interobserver coefficients of variation for all anatomical parameters were > 0.85.

### Comparison of bilateral hip joint parameters

Among the bilateral hip joint parameters, the isthmus width between the right and left sides was significantly different, and its average absolute difference was 1.5 mm (95% confidence interval, 1.3–1.7). There was no significant difference in the other proximal femoral or acetabular parameters (Table 1).

**Table 1:** Comparison of bilateral hip joint parameters of X-ray

Parameter of X-ray	Left	Right	*P* value	AB	95% CI of AB
Offset (mm)	39.2 ± 6.2	39.1 ± 6.4	0.866	2.9 ± 2.5	2.6, 3.3
Height of femoral head (mm)	60.7 ± 6.8	60.8 ± 6.8	0.700	3.3 ± 2.4	3.0, 3.7
T + 20 (mm)	52.8 ± 6.5	52.8 ± 6.8	0.894	3.4 ± 2.9	3.0, 3.7
T + 0 (mm)	31.3 ± 5.3	31.4 ± 5.2	0.625	2.9 ± 2.7	2.5, 3.2
T − 20 (mm)	23.4 ± 4.0	23.5 ± 3.9	0.627	2.3 ± 2.0	2.0, 2.6
Isthmus width (mm)	13.3 ± 2.1	13.6 ± 2.3	0.011	1.5 ± 1.3	1.3, 1.7
Neck-shaft angle (°)	131.9 ± 6.5	132.0 ± 4.8	0.131	3.6 ± 2.6	3.2, 3.9
CFI	4.1 ± 0.7	4.0 ± 0.8	0.052	0.5 ± 0.4	0.4, 0.5
Acetabular abduction angle (°)	41.2 ± 4.0	40.9 ± 4.0	0.103	2.4 ± 1.8	2.2, 2.7
Trochanteric height (mm)	4.0 ± 6.4	3.9 ± 6.4	0.695	2.1 ± 1.8	1.9, 2.4

Data are presented as mean ± standard deviation unless otherwise indicated

*AB* absolute difference, *CI* confidence interval, *CFI* canal flare index

In CT measurement, there was no significant difference in sagittal medullary cavity diameter and anteversion angle between bilateral femurs (Table 2).

**Table 2:** Comparison of bilateral hip joint parameters of CT

Parameters of CT	Left	Right	*P* value	AB	95% CI of AB
Offset (mm)	40.9 ± 4.6	40.1 ± 4.7	0.163	2.1 ± 2.3	1.3, 3.0
Height of femoral head (mm)	44.0 ± 6.6	44.0 ± 6.4	0.435	2.3 ± 1.9	1.7, 3.0
ML-T + 20 (mm)	49.0 ± 6.0	48.0 ± 4.5	0.141	2.7 ± 2.4	1.8, 3.5
AP-T + 20 (mm)	34.7 ± 3.4	34.7 ± 3.7	0.895	1.8 ± 1.0	1.4, 2.1
ML-T + 0 (mm)	29.4 ± 4.5	29.2 ± 3.9	0.539	1.1 ± 1.7	0.5, 1.7
AP-T + 0 (mm)	22.3 ± 3.4	22.2 ± 3.9	0.982	2.0 ± 2.2	1.2, 2.7
ML-T − 20 (mm)	19.2 ± 2.7	18.8 ± 2.7	0.059	1.0 ± 0.7	0.7, 1.2
AP-T − 20 (mm)	19.8 ± 2.8	19.9 ± 2.9	0.595	1.1 ± 0.9	0.9, 1.5
ML-Isthmus width (mm)	13.2 ± 1.9	12.9 ± 2.3	0.566	0.8 ± 0.8	0.6, 1.1
AP-Isthmus width (mm)	13.2 ± 1.9	13.0 ± 2.3	0.253	0.8 ± 0.7	0.5, 1.0
Neck-shaft angle (°)	131.5 ± 4.4	131.2 ± 4.5	0.202	2.7 ± 2.4	1.8, 3.6
ML-CFI	3.7 ± 0.5	3.6 ± 0.4	0.406	0.2 ± 0.3	0.1, 0.3
AP-CFI	2.6 ± 0.4	2.7 ± 0.4	0.121	0.2 ± 0.1	0.1, 0.2
Trochanteric height (mm)	4.7 ± 5.4	4.4 ± 5.8	0.236	2.1 ± 1.8	1.3, 2.8
Anteversion (°)	15.1 ± 7.8	16.1 ± 8.0	0.155	3.0 ± 2.8	2.0, 4.0

Data are presented as mean ± standard deviation unless otherwise indicated

*AB* absolute difference, *CI* confidence interval, *ML* medial–lateral, *AP* anterior–posterior, *CFI* canal flare index

Among all cases in the study, the same model as the prosthetic stem was used in 144 cases and a model different from the prosthetic stem was used in 80 cases. In the group with inconsistent models of the prosthetic stems, we found no significant difference between the two sides (Table 3); we also found no significant AB between the consistent prosthetic stem group and inconsistent prosthetic stem group (Table 4).

**Table 3:** Comparison of absolute difference between left and right sides in the prosthetic stem inconsistency group

Parameter	Left	Right	*P* value	AB	95% CI of AB
Offset	39.9 ± 6.2	40.0 ± 6.4	0.901	2.9 ± 2.4	2.4, 3.5
Height of femoral head	60.7 ± 6.8	60.6 ± 6.4	0.860	3.3 ± 2.6	2.7, 3.9
T + 20	53.1 ± 5.9	53.6 ± 6.7	0.323	3.5 ± 2.8	2.9, 4.1
T + 0	31.9 ± 5.4	32.1 ± 5.4	0.384	2.6 ± 2.1	2.1, 3.1
T − 20	24.0 ± 4.0	24.3 ± 3.8	0.413	2.4 ± 1.9	2.1, 2.9
Isthmus width	13.6 ± 2.1	13.9 ± 2.1	0.060	1.4 ± 1.2	1.1, 1.7
Neck-shaft angle	131.5 ± 4.6	131.7 ± 4.3	0.660	3.7 ± 2.8	3.1, 4.3
CFI	4.0 ± 0.8	3.9 ± 0.8	0.178	0.4 ± 0.4	0.3, 0.5
Acetabular abduction angle	40.9 ± 4.9	41.1 ± 4.5	0.597	2.4 ± 1.8	2.0, 2.8
Trochanteric height	4.9 ± 7.0	4.4 ± 6.8	0.069	1.9 ± 1.7	1.5, 2.3

Data are presented as mean ± standard deviation unless otherwise indicated

*AB* absolute difference, *CI* confidence interval, *CFI* canal flare index

**Table 4:** Comparison of differences between the prosthetic stem inconsistency group and prosthetic stem consistency group

AB	Inconsistency (*n* = 80)	Consistency (*n* = 144)	*P* value
Offset	2.9 ± 2.4	2.9 ± 2.7	0.905
Height of femoral head	3.3 ± 2.6	3.4 ± 2.3	0.918
T + 20	3.5 ± 2.8	3.3 ± 2.7	0.497
T + 0	2.6 ± 2.1	3.0 ± 2.9	0.248
T − 20	2.5 ± 1.9	2.2 ± 2.0	0.295
Isthmus width	1.4 ± 1.2	1.5 ± 1.4	0.563
Neck-shaft angle	3.7 ± 2.8	3.5 ± 2.5	0.466
CFI	0.4 ± 0.5	0.5 ± 0.5	0.123
Acetabular abduction angle	2.4 ± 1.8	2.4 ± 1.8	0.962
Trochanteric height	1.9 ± 1.7	2.3 ± 1.9	0.160

Data are presented as mean ± standard deviation unless otherwise indicated

*AB* absolute difference, *CFI* canal flare index

Considering that the shape of femoral medullary cavity is the same, but there was still different stem size in more than a third of the cases, we analyzed the consistency of stem size between the 1-stage and the 2-stage groups. There were only 20 bilateral stem size different in 96 1-stage cases (20.8%), while there were 60 bilateral stem size different in 128 2-stage cases (48.9%), and the *P* value < 0.001.

There was no significant difference in AB between the male group and female group (Table 5).

**Table 5:** Comparison of differences between the sex

AB	Male (*n* = 135)	Female (*n* = 89)	*P* value
Offset	2.8 ± 2.4	3.0 ± 2.8	0.507
Height of femoral head	3.3 ± 2.4	3.4 ± 2.5	0.848
T + 20	3.4 ± 3.0	3.3 ± 2.4	0.755
T + 0	2.9 ± 2.7	2.9 ± 2.6	0.941
T − 20	2.2 ± 1.8	2.4 ± 2.2	0.382
Isthmus width	1.4 ± 1.3	1.6 ± 1.4	0.133
Neck-shaft angle	3.4 ± 2.5	3.8 ± 2.7	0.172
CFI	0.4 ± 0.4	0.5 ± 0.5	0.124
Acetabular abduction angle	2.5 ± 1.8	2.2 ± 1.9	0.218
Trochanteric height	2.2 ± 1.9	2.0 ± 1.6	0.347

Data are presented as mean ± standard deviation unless otherwise indicated

*AB* absolute difference, *CFI* canal flare index

### Correlation of anatomical parameters

We found no correlation between the AB in the anatomical parameters and age, height, or sex (Table 4). A weak correlation was present between the body weight and neck-shaft angle (NSA) (*R* = 0.230, *P* = 0.013); specifically, an increased body weight was associated with increased asymmetry of the NSA (Table 6).

**Table 6:** Correlation between AB in the morphology of the proximal femoral medullary cavity and demographic data (age, height, and weight)

AB	Age		Height		Weight		Sex	
	*R*	*P*	*R*	*P*	*R*	*P*	*R*	*P*
Offset	− 0.045	0.634	− 0.045	0.628	0.018	0.847	− 0.002	0.984
Height of femoral head	− 0.053	0.573	− 0.059	0.532	0.072	0.440	0.035	0.710
T + 20	− 0.036	0.702	− 0.031	0.737	− 0.154	0.100	0.144	0.122
T + 0	− 0.016	0.862	0.030	0.748	0.012	0.898	0.034	0.719
T − 20	0.072	0.444	− 0.158	0.090	− 0.023	0.810	0.138	0.140
Isthmus width	− 0.065	0.486	0.063	0.504	− 0.027	0.777	− 0.053	0.570
Neck-shaft angle	0.006	0.67	− 0.098	0.293	0.230	0.013	0.002	0.986
CFI	− 0.034	0.946	0.040	0.668	− 0.126	0.177	− 0.027	0.772
Acetabular abduction angle	− 0.094	0.317	− 0.052	0.577	0.031	0.743	0.081	0.389
Trochanteric height	− 0.028	0.766	0.015	0.872	0.151	0.106	0.020	0.831

*CFI* canal flare index, *AB* absolute difference;

Among the anatomical parameters, the height of the femoral head had weak negative correlations with T − 20 (*R* = − 0.204, *P* = 0.029), the isthmus width (*R* = − 0.203, *P* = 0.030), and the acetabular abduction angle (*R* = − 0.183, *P* = 0.050); T + 20 was weakly correlated with T − 20 (*R* = 0.239, *P* = 0.010); T + 0 had a general correlation with T − 20 (*R* = 0.402, *P* < 0.001), a weak negative correlation with the height of the femoral head (*R* = − 0.204, *P* = 0.029), and a weak correlation with T + 20 (*R* = 0.239, *P* = 0.010); and the difference in the isthmus width had a general correlation with the difference in the CFI (*R* = 0.604, *P* < 0.001). (See Table 7).

**Table 7:** Correlation between absolute differences in medullary cavity morphology of the proximal femur (positive results)

AB	Variable	*R* value	*P* value
Height of femoral head	T-20	− 0.204	0.029
	Isthmus width	− 0.203	0.030
	Acetabular abduction angle	− 0.183	0.050
T + 20	T-20	0.239	0.010
T + 0	T-20	0.402	< 0.001
T − 20	Height of femoral head	− 0.204	0.029
	T + 20	0.239	0.010
	T + 0	0.402	< 0.001
Isthmus width difference	Height of femoral head	− 0.203	0.030
	CFI difference	0.604	< 0.001
CFI difference	Isthmus width difference	0.559	< 0.001
Acetabular abduction angle difference	Height of femoral head	− 0.183	0.050

*CFI* canal flare index, *AB* absolute difference

### Postoperative indicators

The average leg length difference was (3.2 ± 3.7) mm. There was no significant difference between the 1-stage group (3.0 ± 3.9) mm and 2-stage group (3.3 ± 4.0) mm (*P* = 0.369). The leg length difference > 5 mm was defined as the leg length discrepancy (LLD). There were 62 patients with LLD, even 18 of them were larger than 10 mm. There was no significant difference between the 1-stage and the 2-stage groups (*P* = 0.365).

The results showed that there was no significant difference in postoperative offset between left (46.4 ± 7.2) mm and right (46.4 ± 7.0) mm (*P* = 0.882). However, there were significant differences in AB between male (2.3 ± 1.8) and female (1.8 ± 1.6), the *P* value was 0.021.

## Discussion

The success of THA depends on accurate matching of the femoral morphology and prostheses [5]. Therefore, precise measurement of the hip joint anatomical parameters is crucial. An effective preoperative plan can greatly reduce the operation time and difficulty, enhance the curative effect of the operation, and reduce the need for a revision operation. Among patients with hip joint deformities such as those induced by femoral neck fracture, DDH, or coxa plana, preoperative template measurements are often difficult. The normal anatomical structure of the uninjured side is usually used for the preoperative plan, and the hip joint morphology of the operation side is reconstructed using quantitative hip joint data to correct the hip joint morphology with any deformities.

A preliminary preoperative plan can be established using this method, and such a plan is safer and more effective than measurement of the injured limb with intraoperative planning. Of course, this method relies on the morphological symmetry of the bilateral hip joints in the normal population, or the difference in baseline data can be ignored. In addition, an understanding of the baseline differences in the bilateral hip joints can help to understand hip joint deformities and disease progression.

In this study, the parameters of the bilateral hip joints were symmetrical, and there was no statistically significant difference in any parameter except the isthmus width. We believe that the difference in the isthmus width may have been associated with the varying degrees of degenerative osteoporosis caused by the inconsistent degree of bilateral lesions in the patients who required hip arthroplasty on both sides because of bilateral disease.

In the 30 cases of CT data we studied, there was no bilateral difference in sagittal plane and anteversion. The application of CT is a preoperative plan, which can effectively understand the three-dimensional structure of the femur [16, 20]. It is very useful for patients with three-dimensional structural deformities of the hip, such as DDH, or patients with a history of proximal femoral osteotomy. Reconstruction of the hip joint position is often difficult. It should be noted that there are not only great changes in neck-shaft angle and offset in patients with deformity [13, 21], but also the anteversion of femur has changed a lot, Liu et al.[10] showed the femoral anteversion of DDH patients ranged from − 3.2° to 58.2° with an average of 22.2°. However, our study showed that the anteversion of femur was an average of 15.1° ± 7.8 in the left and 16.1° ± 8.0 in the right. Previous study suggested to use the assembled prosthesis stem to match the shape of medullary cavity and restore the anteversion [14], and this is also the method we use in surgery.

In our study, there was no significant difference in the bilateral acetabular abduction angle, which was not related to the proximal femoral parameters. When the acetabular destruction is severe or the normal anatomy is difficult to distinguish, the uninjured side can be used for reference.

Further analysis showed that there was no difference in the parameters of the bilateral proximal femurs in the patients with inconsistent prosthetic stems; while the 2-stage group seemed to be more likely in using inconsistent prosthetic stems. This finding might have been associated with the difference in the intraoperative osteotomy position, the recovery of the leg length during surgery, and the surgeon’s empirical selection of the prosthesis.

In our study, there were no difference in LLD and AB of offset between the prosthetic stem inconsistency and consistency group after the surgery. The use of an inconsistent model of the prosthetic stems did not affect the difference in the anatomical parameters of the bilateral hip joints [24].

In the data of X-ray, a correlation was present among the T + 20, T + 0, and T − 20 planes, indicating that the degree of asymmetry in the proximal femur was not affected by one plane but was instead correlated with adjacent planes. Noble et al. [12] and Sugano et al. [17] also found that the anatomical parameters of the femoral medullary cavity were highly correlated with adjacent segments. Young et al. [24] found that the planes with asymmetry in the coronal position were also asymmetric in the sagittal position, indicating that bilateral asymmetry did not only appear in an orthogonal plane, when asymmetry occurred in one plane, asymmetry was also present in its orthogonal plane. However, in our CT samples, we did not find any correlation between coronal and sagittal planes and, therefore, were not included in the table of correlation analysis which only showed positive results. We think it is related to the AB values were numerical small and the small CT sample size.

In addition to the weight had affected the AB of NSA, demography had no effect on difference of bilateral femur. This conclusion has been confirmed by many previous studies. Ollivier et al. [15] suggested that there was no correlation between gender and hip anatomy results. Young et al. [24] found that the proximal femoral anatomy was bilaterally symmetrical, and this symmetry was not affected by the demographic data. However, the effect of demography on the morphology of femoral medullary cavity is still controversial [19]. Edwards et al. [3] showed that the average of lateral center edge angle, minimum neck width and neck length differed between sex. The difference in conclusion may be due to the different sample population.

LLD is a common complication of THA, which may cause short-term hip pain and discomfort that result in lameness, compensatory pelvic tilt or scoliosis, and even aseptic loosening of the prosthesis in the long term [4, 11]. Previous literature has shown that it is clinically acceptable to have unequal leg length of less than 10 mm in terms of imaging [1]. In our study, more than 1/4 patients had LLD after bilateral hip replacement, even 18 of them were larger than 10 mm. This study shows that the 2-stage bilateral THA is more likely to have different types of prosthesis stem than the 1-stage bilateral THA, however, this factor has no significant impact on difference of leg length.

A previous study indicated that the tip of the greater trochanter was an average of 8 mm higher than the femoral head center (range, 4–24 mm) [7]. In this study, the trochanter height on both sides is consistent, which allowed us to use it as a reference for leg length reconstruction on the injured and uninjured sides.

The offset is an important anatomical parameter that needs to be recovered in THA. A small offset can lead to insufficient local muscle tension and gluteal muscle weakness and can even cause hip dislocation in severe cases [6, 9]. When the offset is small and the soft tissue tension is insufficient during surgery, it is often adjusted by increasing the neck length, which may easily lead to prolongation of the injured limb. In this study, the bilateral offset was symmetric and independent of demographic factors such as age and sex. Therefore, the contralateral offset is a consideration for structural reconstruction of the hip joint. Difficult recovery of the offset is sometimes related to the insufficient selective NSA for prosthetic stem. Young et al. [24] reported that the asymmetry of the femoral neck was positively correlated with the body height [24]. In this study, the bilateral difference in the NSA had a linear relationship with the body weight and medullary isthmus width. When the difference in the medullary cavity width increased, the difference in the bone density increased, which may have caused the lower bone density and loading ability. At the same time, with the increase in body weight, the femoral moment load became excessively large and the NSA was reduced.

This study had the following limitations. First, this study is mainly based on two-dimensional morphological measurement, and not much three-dimensional data are included, which cannot measure the three-dimensional information of proximal femoral medullary cavity and acetabulum in detail. Yi et al. [23] showed that there was a significant negative correlation between offset and NSA based on CT scans. Although X-ray film is still the most commonly used for preoperative in THA, three-dimensional data can be applied to design better ethnic-specific THA prostheses and preoperative template in some difficult cases. Second, the patients included in this study were only Asian patients and that may limit the impact of our findings. Previous literature has demonstrated differences in medullary cavity morphology between the geographic origin of Asian and European [22].

Our study supports the assumption of bilateral symmetry of the hip joints. The lack of a significant difference between the two sides theoretically confirms that it is feasible to implement hip joint reconstruction of the injured side using the hip morphology of the uninjured side. In addition, the morphological changes of the proximal femoral medullary cavity are not present in a single plane but are instead affected by multiple planes.

## Data Availability

All data included in this study are available upon request by contact with the corresponding author.
